# First human infection of avian influenza A(H5N6) virus reported in Lao People's Democratic Republic, February–March 2021

**DOI:** 10.1111/irv.12934

**Published:** 2021-11-10

**Authors:** Bounthanom Sengkeopraseuth, Kim Carmela Co, Phetdavanh Leuangvilay, Joshua A. Mott, Boungnasith Khomgsamphanh, Virasack Somoulay, Reiko Tsuyuoka, May Chiew, Pakapak Ketmayoon, Joyce Jones, Elizabeth Pusch, Yunho Jang, John Barnes, Charles Todd Davis, Phouvong Phommachanh, Bouaphanh Khamphaphongphane, Sonja J. Olsen, Phonepadith Xangsayyarath

**Affiliations:** ^1^ National Center for Laboratory and Epidemiology, Department of Communicable Disease Control Ministry of Health Vientiane Lao PDR; ^2^ World Health Organization Vientiane Lao PDR; ^3^ U. S. Centers for Disease Control and Prevention Atlanta Georgia USA; ^4^ Epidemiology Unit Provincial Health Department Luang Prabang Lao PDR; ^5^ National Animal Health Laboratory, Department of Livestock and Fisheries Ministry of Agriculture and Forestry Vientiane Lao PDR

**Keywords:** H5N6 subtype, human infection, influenza A virus, Laos, poultry, rapid response team

## Abstract

In March 2021, Lao People's Democratic Republic (Laos) reported an avian influenza A(H5N6) virus infection in a 5‐year‐old child identified through sentinel surveillance. This was the first human A(H5N6) infection reported outside of China. A multidisciplinary investigation undertook contact tracing and enhanced human and animal surveillance in surrounding villages and live bird markets. Seven Muscovy ducks tested positive for highly pathogenic avian influenza A(H5N6) viruses. Sequenced viruses belonged to clade 2.3.4.4h and were closely related to viruses detected in poultry in Vietnam and to previous viruses detected in Laos. Surveillance and coordinated outbreak response remain essential to global health security.

## 1 INTRODUCTION

From 2014 to 2021, highly pathogenic avian influenza (HPAI) A(H5N6) viruses have been circulating in poultry in Asia and Africa.^1^ Every detection presents a risk to exposed humans and a pandemic threat. As of October 2021, a total of 47 laboratory‐confirmed cases of human infection with influenza A(H5N6) virus including 24 deaths have been reported to WHO since 2014.^1^, ^2^ In March 2021, a human infection with avian influenza A(H5N6) virus was identified in a 5‐year‐old boy in Luang Prabang province in Lao People's Democratic Republic (Laos) through routine surveillance for influenza‐like illness. Here, we report the findings from a multidisciplinary rapid response investigation.

## 2 OUTBREAK INVESTIGATION

Staff from the National Center for Laboratory and Epidemiology (NCLE), Ministry of Health, Laos, and the WHO deployed to Luang Prabang on March 14, 2021, to support the provincial health department. Response objectives were to identify close contacts of the case through house‐to‐house interviews; engage with village authorities and school administrators to report any unusual clusters of respiratory illnesses; retrospectively review ILI and SARI data from six health facilities for the period from February 1 to March 16, 2021; and, prospectively, increase specimen collection for respiratory illnesses in three hospitals, including the military hospital where the case was identified. The investigation focused on four villages, including the village of residence of the case and three neighboring villages. The team also coordinated with the National Animal Health Laboratory, Department of Livestock and Fisheries, and the provincial agriculture and forestry department to enhance surveillance in domestic poultry and waterfowl in the four villages, and in the two live bird markets.

A suspected human case of avian influenza A(H5N6) virus infection was defined as a temperature ≥ 38°C and respiratory symptoms (cough, shortness of breath, or difficulty breathing) with onset on or after February 1 in a person who lived in one of the four villages (Vang Ngeun, Phouxangkham, Donekeo, and Houaiyen). A close contact was defined as a person who was within 1 m and cared for, touched, or spoke to a person confirmed to have avian influenza A(H5N6) virus infection (without appropriate personal protective equipment) from 1 day before through 14 days after the case patient's illness onset. Close contacts had specimens collected at the time of interview and were monitored for symptoms for 14 days.

**FIGURE 1:**
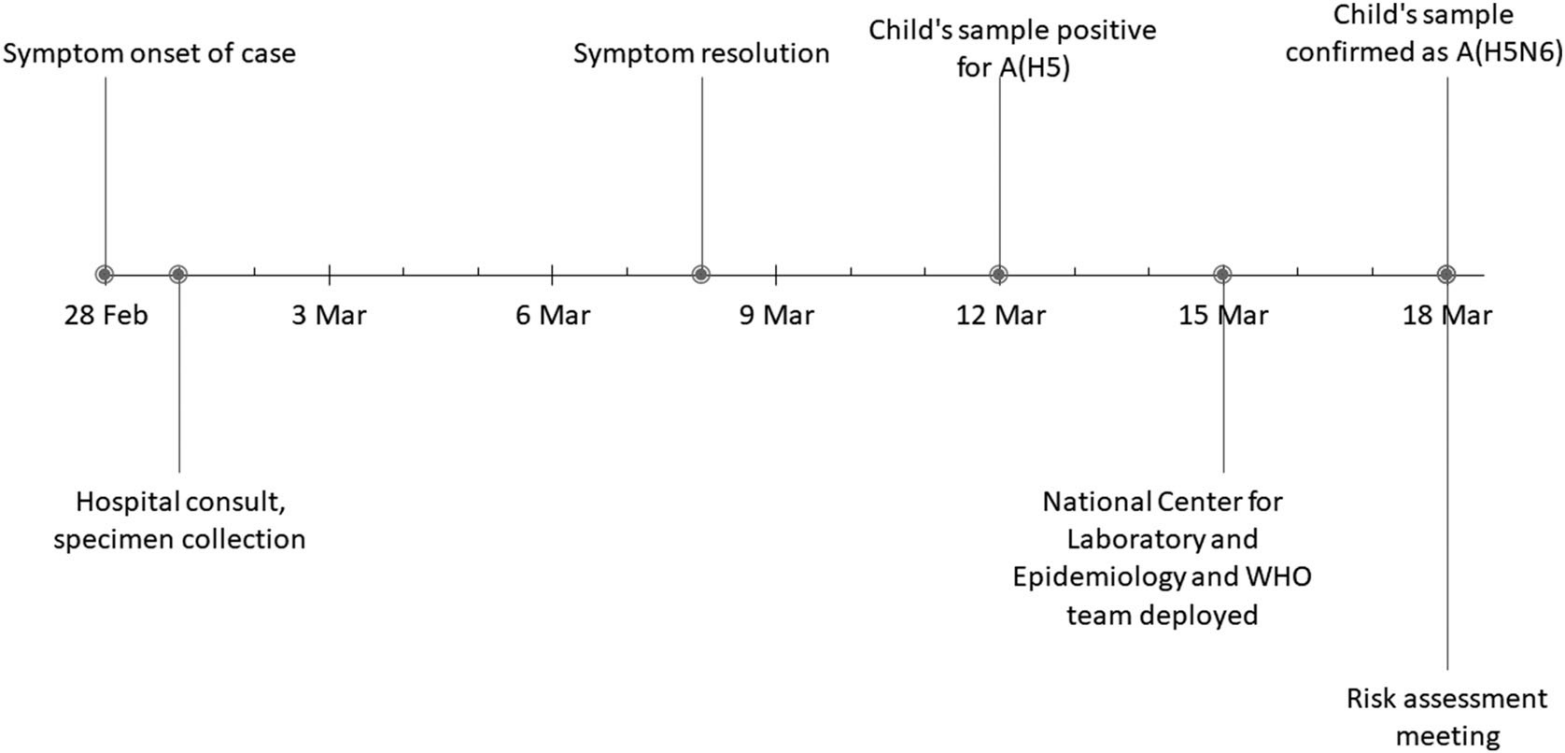
Timeline of events by day related to the human infection of avian influenza A(H5N6) virus in Lao People's Democratic Republic, February–March 2021

The child became ill on February 28, 2021, with a temperature of 37.5°C and cough and sought care at the outpatient department of the nearby military hospital on March 1 (Figure 1). Oropharyngeal and nasopharyngeal swabs were collected as part of ILI sentinel surveillance, and the child was prescribed amoxicillin and antipyretics. The child remained at home until March 8 when signs and symptoms resolved, and he returned to school. On March 12, the Lao NCLE confirmed that the child's sample was positive for avian influenza A(H5) virus by real‐time reverse transcription‐polymerase chain reaction (RT‐PCR), and the child, though without signs or symptoms at that time, was hospitalized as a precautionary measure. The three other household family members remained healthy, and samples collected on March 13 were negative for influenza viruses. Under the International Health Regulations (IHR 2005), the Ministry of Health notified WHO on March 13. On March 18, further tests by real‐time RT‐PCR confirmed an avian influenza A(H5N6) virus.

The child's village (Vang Ngeun) included a population of 1822 people in 378 families. Interviews identified 71 close contacts of the child: 11 health care workers, 3 household family members, 25 people in nearby households, and 32 people (2 teachers and 30 students) in the kindergarten where the infected child went to school. None reported any recent or current illness. The infected child reported regular contact with both chickens and ducks in his yard and in neighboring yards.

A review of surveillance for ILI and SARI in the three hospitals in the province found no observed increase in cases in January–February 2021 compared with 2020. The enhanced case finding from March 16 through April 8 identified a total of 139 ILI and 2 SARI cases. Five persons tested positive for influenza A(H3) viruses, whereas 134 were negative for influenza viruses.

The multidisciplinary team conducted a real‐time risk assessment for this event and concluded that there was a high risk of undetected infection in poultry due to past reports of poultry illness and death, and a moderate risk of additional human cases.

In the child's village, 172 of 3700 chickens and ducks were reported to have died, including ducks in the child's household and in the five neighboring households during the 20 days preceding the child's illness. The child's household raised six chickens and one duck; one chicken was reportedly lethargic with weight loss and was slaughtered and consumed by the family on March 3, 2021 (after the child was ill). In Vang Ngeun village, 20 cloacal swabs, 6 oropharyngeal swabs, and 17 environmental samples were collected and submitted to National Animal Health Laboratory; all tested negative for avian influenza viruses. In Phouxangkham village (next to Vang Ngeun village), where there were reports of bird deaths in September and October 2020, 7 of 90 Muscovy duck (*Cairina moschata domestica*) cloacal swabs taken for this investigation tested positive for avian influenza A(H5N6) viruses. In the other villages, 161 specimens and 10 specimens from ducks at a nearby hatchery all tested negative for influenza A viruses.

The Department of Livestock and Fisheries (DLF) performed culling, disinfection, and disposal within the poultry markets of Phouxangkham village where avian influenza A(H5N6) virus was confirmed in the duck and in the child's village. There was no compensation provided for poultry that were culled. Enhanced poultry surveillance was also established in a 5‐km radius around these villages for 14 days. The DLF established checkpoints in order to disinfect vehicles and cargo and to control movement of poultry for 14 days. Finally, a public awareness campaign was initiated to enhance disinfection of premises where poultry are kept; biosafety practices when visiting farms; isolation of poultry flocks from outside environments and wild birds; and to raise awareness of the signs and symptoms of avian influenza in poultry.

**FIGURE 2:**
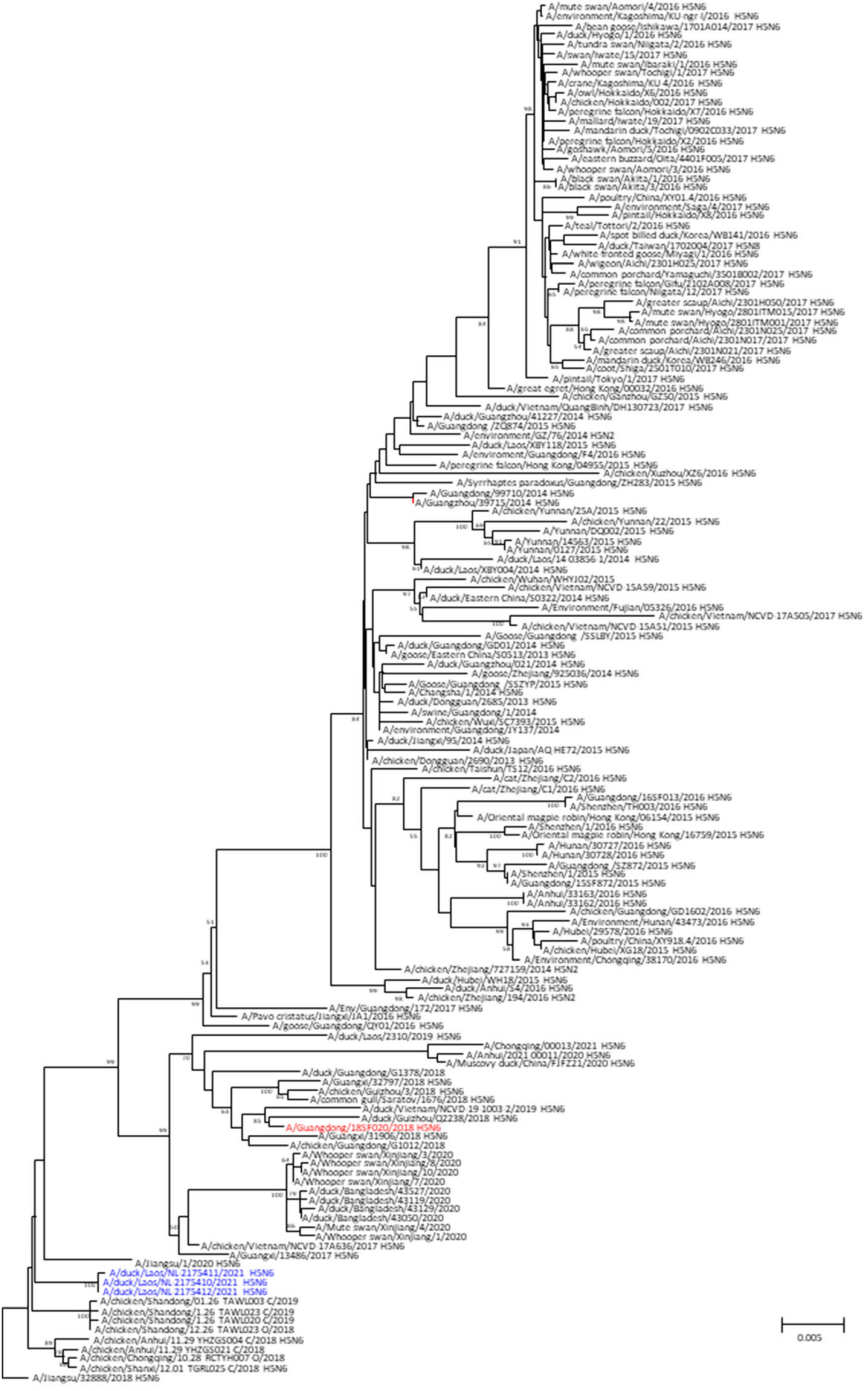
Phylogenetic tree of the hemagglutinin genes of representative clade 2.3.4.4h influenza A(H5N6) viruses. Samples sequenced for this study are in blue font. The nearest pre‐pandemic candidate vaccine virus is shown in red font. Numbers above and below branches represent bootstrap values after 1000 replicates following maximum likelihood analysis

The human and bird specimens were sent to the WHO Collaborating Center at the Centers for Disease Control (CDC), USA, for further characterization. Real‐time RT‐PCR testing of the human specimen yielded a high cycle threshold (>36) for HPAI A(H5) and virus could not be recovered in cells or eggs. CDC generated codon complete gene sequences for three viruses collected from ducks. The three viruses were all clade 2.3.4.4h HPAI A(H5N6) viruses closely related to viruses detected in poultry in Vietnam and previous viruses detected in Laos (Figure 2). CDC also isolated the viruses in eggs and confirmed they were antigenically related to a recently developed WHO candidate vaccine virus (A/Guangdong/18SF020/2018‐like; IDCDC‐RG65A).

## 3 CONCLUSIONS

A child identified through influenza sentinel surveillance in Laos was found to have an avian influenza A(H5N6) virus infection; the outbreak investigation identified exposure of the child to sick poultry in the child's household and infected ducks in a neighboring village suggesting that the infection was likely acquired from infected birds. No subsequent human to human transmission was identified in the tracing of close contacts. This is the first time an avian influenza A(H5N6) virus has been detected in humans in Laos, and the first human infection reported outside of China. However, infections in humans have been detected in Southern China and in poultry in China, Laos, and Vietnam.^3^, ^4^

Seventy percent of reported human infections with HPAI A(H5N6) viruses have been in persons who died,^2^ but surveillance likely preferentially detects those with more severe illness. The infection in the child in Laos was mild and detected through routine surveillance for ILI. This finding highlights the importance of maintaining ongoing ILI and SARI surveillance, and laboratory capacity to identify avian influenza subtypes, cornerstones of the WHO's Global Influenza Surveillance and Response System.^5^ It also reinforces the need for regular and close coordination between human and animal health authorities. Despite recent poultry mortality, the detection of the virus in poultry occurred during the multidisciplinary outbreak investigation. In Southeast Asia, where much of the population is rural and exposed to poultry,^6^ the likelihood of exposure to avian viruses remains elevated. The recent mild infection of avian influenza A(H9N2) virus in a child in Cambodia, also detected through ILI surveillance, further demonstrates the value of sentinel surveillance.^7^

Since the introduction of HPAI A(H5N1) virus into Southeast Asia in 2004, rapid response investigations by multidisciplinary teams have become more common. In this situation, One Health coordination and established laboratory capacity produced an efficient response to a signal identified in routine human surveillance. Zoonotic influenza viruses remain a substantial pandemic threat.^8^ This outbreak demonstrates that activities to support the International Health Regulation's core capacities for sentinel and event‐based surveillance are essential to public health and pandemic preparedness. Further, coordinated outbreak response, rapid reporting, and virus sharing to the World Organisation for Animal Health (OIE) and WHO, as well as genetic and antigenic characterization of novel viruses to develop candidate vaccine viruses, remain critical for global health security.

### AUTHOR CONTRIBUTIONS

**Bounthanom Sengkeopraseuth:** Conceptualization; data curation; investigation; methodology; resources; supervision. **Kim Co:** Conceptualization; data curation; formal analysis; funding acquisition; investigation; methodology; project administration; supervision. **Phetdavanh Leuangvilay:** Conceptualization; investigation; methodology; project administration. **Joshua Mott:** Conceptualization; data curation; formal analysis; funding acquisition; project administration; resources. **Boungnasith Khomgsamphanh:** Conceptualization; investigation; methodology; project administration. **Virasack Som Oulay:** Conceptualization; funding acquisition; investigation; methodology; project administration. **Reiko Tsuyuoka:** Conceptualization; funding acquisition; investigation; methodology; project administration; resources; supervision. **May Chiew:** Conceptualization; data curation; formal analysis; funding acquisition; investigation; methodology; project administration. **Pakapak Ketmayoon:** Conceptualization; investigation; methodology; project administration; resources; supervision. **Joyce Jones:** Data curation; formal analysis. **Elizabeth Pusch:** Data curation; formal analysis; methodology. **Yunho Jang:** Data curation; formal analysis. **John Barnes:** Data curation; formal analysis; methodology; project administration. **C. Todd Davis:** Formal analysis; funding acquisition; methodology. **Phouvong Phommachanh:** Conceptualization; data curation; formal analysis; funding acquisition; investigation; methodology; project administration. **Bouaphanh Khamphaphongphane:** Conceptualization; data curation; formal analysis; funding acquisition; investigation; methodology; project administration. **Sonja Olsen:** Conceptualization; formal analysis; funding acquisition; methodology. **Phonepadith Xangsayarath:** Conceptualization; formal analysis; funding acquisition; investigation; methodology; project administration.

### PEER REVIEW

The peer review history for this article is available at https://publons.com/publon/10.1111/irv.12934.

## Data Availability

The data that support the findings of this study are the property of the Government of Lao People's Democratic Republic. Any further availability of data would be subject to applicable governmental approvals.
